# Ethnic disparities in mortality and group-specific risk factors in the UK Biobank

**DOI:** 10.1371/journal.pgph.0001560

**Published:** 2023-02-23

**Authors:** Kara Keun Lee, Emily T. Norris, Lavanya Rishishwar, Andrew B. Conley, Leonardo Mariño-Ramírez, John F. McDonald, I. King Jordan

**Affiliations:** 1 School of Biological Sciences, Georgia Institute of Technology, Atlanta, GA, United States of America; 2 Integrated Cancer Research Center, School of Biological Sciences, Georgia Institute of Technology, Atlanta, GA, United States of America; 3 IHRC-Georgia Tech Applied Bioinformatics Laboratory, Atlanta, GA, United States of America; 4 National Institute on Minority Health and Health Disparities, National Institutes of Health, Bethesda, MD, United States of America; International Institute for Population Sciences, INDIA

## Abstract

Despite a substantial overall decrease in mortality, disparities among ethnic minorities in developed countries persist. This study investigated mortality disparities and their associated risk factors for the three largest ethnic groups in the United Kingdom: Asian, Black, and White. Study participants were sampled from the UK Biobank (UKB), a prospective cohort enrolled between 2006 and 2010. Genetics, biological samples, and health information and outcomes data of UKB participants were downloaded and data-fields were prioritized based on participants with death registry records. Kaplan-Meier method was used to evaluate survival differences among ethnic groups; survival random forest feature selection followed by Cox proportional-hazard modeling was used to identify and estimate the effects of shared and ethnic group-specific mortality risk factors. The White ethnic group showed significantly worse survival probability than the Asian and Black groups. In all three ethnic groups, endoscopy and colonoscopy procedures showed significant protective effects on overall mortality. Asian and Black women show lower relative risk of mortality than men, whereas no significant effect of sex was seen for the White group. The strongest ethnic group-specific mortality associations were ischemic heart disease for Asians, COVID-19 for Blacks, and cancers of respiratory/intrathoracic organs for Whites. Mental health-related diagnoses, including substance abuse, anxiety, and depression, were a major risk factor for overall mortality in the Asian group. The effect of mental health on Asian mortality, particularly for digestive cancers, was exacerbated by an observed hesitance to answer mental health questions, possibly related to cultural stigma. C-reactive protein (CRP) serum levels were associated with both overall and cause-specific mortality due to COVID-19 and digestive cancers in the Black group, where elevated CRP has previously been linked to psychosocial stress due to discrimination. Our results point to mortality risk factors that are group-specific and modifiable, supporting targeted interventions towards greater health equity.

## Introduction

Despite the progress made in improving mortality rate, life expectancy, and disease survival outcomes in the last century, health disparities between various population groups persist and remain a major global health issue. Mortality rates are a key indicator of a population’s overall health status and have been long tracked and documented in countries including the United Kingdom (UK) and the United States (US) since 1901 and 1890, respectively [1, 2]. While the mortality gap between race and ethnicity groups have narrowed, the decreasing trend has leveled off in recent years: mortality disparity continues to exist and is variously complex across different populations, geographies, and mortality causes [2, 3]. In particular, the ongoing pandemic of Coronavirus disease 2019 (COVID-19) exemplifies the profound adverse effects of disparity, demonstrated by the disproportionate burden and number of deaths among high-risk and medically underserved racial and ethnic minority groups [4, 5].

Both environmental and genetic factors, with increasing evidence for interaction between environment and genetics through epigenetic mechanisms, have been cited as contributors of health disparities [6–10]. Specifically, the role of differential socioeconomic status (SES), access to healthcare, and allostatic load in mortality disparities had been previously cited as significant risk factors for disparity in mortality [6, 11, 12]. However, studies that explore the potential contributions of many other mortality risk factors together, including health behaviors, medical histories, and dietary factors, are scarce. Moreover, much of health disparity research currently focuses on describing the areas and sizes of disparity, by testing a stratified population in a single model with race and ethnicity as a predictor, and therefore lack information on underlying risk factors specific to each group. In order to effectively reduce disparity, it is crucial to first understand the key contributors to overall and prevalent-cause mortalities specific to each ethnicity, which can be taken to suggest targeted interventions with the greatest likelihood of impact for each group.

The aim of this study was to investigate the disparity in mortality patterns and identify important phenotypic risk factors for the three largest ethnic groups in the UK by using the United Kingdom Biobank (UKB) prospective cohort study, a National Health Service initiative for building health registry of 500,000 people aged between 40 and 69 from 2006 to 2010 [13]. By leveraging UKB’s comprehensive data spanning physical measures, lifestyle, blood and urine biomarkers, imaging, genetic, and linked medical and death registry records, coupled with group-specific feature selection methods and survival models, we aimed to identify top mortality risk factors that are measurable and potentially modifiable [14]. We hope that these findings can inform precise strategies for each ethnicity with the goal of improving mortality for all.

## Materials and methods

### Ethics statement

Ethics approval for the UKB was obtained from the North West Multi-centre Research Ethics Committee (MREC) for the United Kingdom, the Patient Information Advisory Group (PIAG) for England and Wales, and the Community Health Index Advisory Group (CHIAG) for Scotland (see https://www.ukbiobank.ac.uk/learn-more-about-uk-biobank/about-us/ethics).

### UK biobank data & preparation

Data-fields for each individual in the study cohort were downloaded on 3/18/2021 from UKB. With ~6% of the study cohort having experienced death, we prioritized our study on data-fields applicable to the individuals who had death registry records: we applied a series of automated and manual filters to the data-fields, starting with keeping fields that had values for individuals with death records (2,512 non-unique data-fields). The second filter was to keep data-fields with ≥80% record completeness (*n* = 326), followed by manual filtering to merge related records and transform field responses as needed. Diagnosis fields (field 41202) were grouped based on ICD-10 blocks, and operation fields (field 41272) were grouped according to the Chapters as defined in the UKB Data Showcase. For each of these fields, we transformed the binary occurrence of a diagnosis or operation for an individual into a count of the ICD-10 block or operation chapter to not be too granular when defining features for our models. The final set of 240 data-fields was used as features for our model selection. Further, all data-fields were categorized in accordance with UKB’s “Primary Category of Origin” (S1 Fig).

### Genetic ancestry inference

GA inference was performed to estimate six ancestry proportions (African, European, East Asian, Central Asian, South Asian, and West Asian) for 477,205 UKB participants using their whole genome genotypes (WGG) characterized using the UK Biobank Axiom Array or the UK BiLEVE Axiom Array [15]. Participant WGG were merged and harmonized with whole genome sequence (WGS) data from global reference populations, the 1000 Genomes Project (1KGP) and the Human Genome Diversity Project (HGDP), as indicated in S1 Table [16–18]. WGG and WGS variant data were merged to include variants present in all three datasets with variant strand flips and identifier inconsistencies corrected and were filtered for sample missingness <5% and a minor allele frequency >1%. The merged genome variant data set was pruned for linkage disequilibrium using PLINK v2 with ‘—indep pairwise 100 10 0.05’ [19]. Principal component analysis (PCA) on genome variant dataset followed using the FastPCA program implemented in PLINK v2 (S2 Fig) [20]. Finally, genome-wide GA inference that analyzes the PCA data from global reference populations and non-reference individuals with non-negative least squares (NNLS) was implemented using the Rye algorithm as previously described [21].

### Feature selection

Top feature selection of mortality risk factors for each of the three ethnic groups was based on combined ranking of results from Cox proportional-hazard (Cox-PH) modeling and random survival forest model using survival and randomForestSRC package, respectively, in R version 3.6.1 [22, 23]. Univariable Cox-PH model evaluated the importance of each variable for the ethnic groups and their overall or cause-specific mortality predictions based on concordance or Harrell’s C-index. Follow-up times were calculated as the time between UKB study enrollment and either death (OS = 1) or last data download in years. Most common level of categories or median numerical values in each group were set as reference level with age at diagnosis included as fixed covariate in the Cox-PH models. In addition, random forest models were also constructed using *rfsrc* function (ntree = 1000, nsplit = 10, nodesize = 15) with imputation allowed for missing numerical values based on random forest or *impute*.*rfsrc*. Thereafter, variables were ranked on importance based on minimal depth using the *var*.*select* function. Feature selection was using random forest minimal depth method and optimal number of features in the model was validated using cross validation (CV), where average C-index for random survival forest model for each top feature set (size increasing by 5) across 5 repeats of 5-fold CV were calculated. Optimal number of features for multivariable model was based on the minimal numbers of features yielding C-index within 0.5% of the max C-index across the 5 repeats (S4 Fig). The rankings from Cox-PH and random forest were averaged to provide the final list of top mortality risk factors for multivariable modeling.

### Survival modeling analysis

Multivariable Cox-PH survival models were constructed for each ethnic group using all of Asian and Black participant data (Table 1), while 10,000 subsampled participants from the White ethnic group (random sampling without replacement; 20,000 for lung and bronchus cancer model to ensure >100 events) were used for modeling. Optimal seed for random subsampling were selected that preserves the mortality proportions in the three enrollment age categories (≤ 50, 51–65, and ≥ 66) and sex categories of the entire White cohort (Table 1). In addition to overall mortality models, cause-specific mortality models were subsequently constructed for selected causes of death based on relative frequency and standardized residuals of chi-squared test: COVID-19 (Black), ischemic heart disease (Asian), lung and bronchus cancers (White), and digestive cancers (all). General model construction started with the optimal number of selected risk factors determined in the feature selection step and were reduced using backward stepwise selection method based on Akaike information criterion. Age at enrollment categories, sex, and six GA proportions were included in the model as fixed covariates. Proportional hazards assumption was also checked using the *cox*.*zph* function in the survival R-package and the covariates were dropped or stratified when violating the assumption. Finally, significant risk factors in final model were rescaled and transformed as necessary, especially for blood biochemistries with expected ranges or threshold for normal (S2 Table).

**Table 1:** Characteristics of the three ethnic group study cohorts in the UK Biobank (UKB).

Characteristic	Full Cohort	Asian group	Black group	White group
	*(N = 490*,*610)*	*(n = 9*,*877)*	*(n = 8*,*038)*	*(n = 472*,*695)*
Sex (% in ethnic group):				
*Female*	266,650 (54.35)	4,582 (46.39)	4,639 (57.71)	257,429 (54.46)
*Male*	223,960 (45.65)	5,295 (53.61)	3,399 (42.29)	215,266 (45.54)
Age of enrollment (%):				
*≤ 50*	127,103 (25.91)	4,121 (41.72)	4,019 (50.00)	118,963 (25.17)
*51–65*	290,698 (59.25)	4,762 (48.21)	3,337 (41.52)	282,599 (59.78)
*≥ 66*	72,809 (14.84)	994 (10.06)	682 (8.48)	71,133 (15.05)
Overall survival (OS = 1 or dead %)	32790 (6.68)	458 (4.64)	335 (4.17)	31,997 (6.77)
Cause-specific (% in dead):				
*Digestive neoplasm*^*†*^	5107 (15.17)	42 (9.17)	45 (13.43)	5020 (15.69)
*Ischemic heart disease*^*†*^	3513 (10.71)	102 (22.27)	27 (8.06)	3384 (10.58)
*COVID-19*^*†*^	623 (1.90)	17 (3.71)	26 (7.76)	580 (1.81)
*Respiratory/intrathoracic organ (bronchus & lung) neoplasms*^*†*^	2943 (8.98)	19 (4.15)	17 (5.07)	2907 (9.09)

† Primary reason of death coded in ICD10 were used to define cause-specific mortality cohorts for primary malignant neoplasms of digestive organs (C15-26), ischemic heart disease (I20-25), COVID-19 (U7.1 &U 7.2), and primary malignant neoplasm of bronchus and lung (C34)

### Ethnicity interaction analysis

In order to assess potential differential effects of mortality risk factors across the three ethnic groups, Cox-PH models of pooled samples Black, Asian, and randomly subsampled White participants in UKB for all-cause and digestive cancers mortality were constructed. These models were subjected to same model selection and checking steps as previously described, starting with a full model containing all GA-and-mortality specific selected features. For significant predictors in the final model (α = 0.05), interaction terms with ethnicity (Asian*RiskFactor and Black*RiskFactor, with White as reference) were added to evaluate for significant interaction between ethnicity and mortality risk factors [24]. Forest plot of interaction results were plotted using the *plot_model* function in the sjPlot R-package.

## Results

### Genetic ancestry and mortality patterns

**Fig 1:**
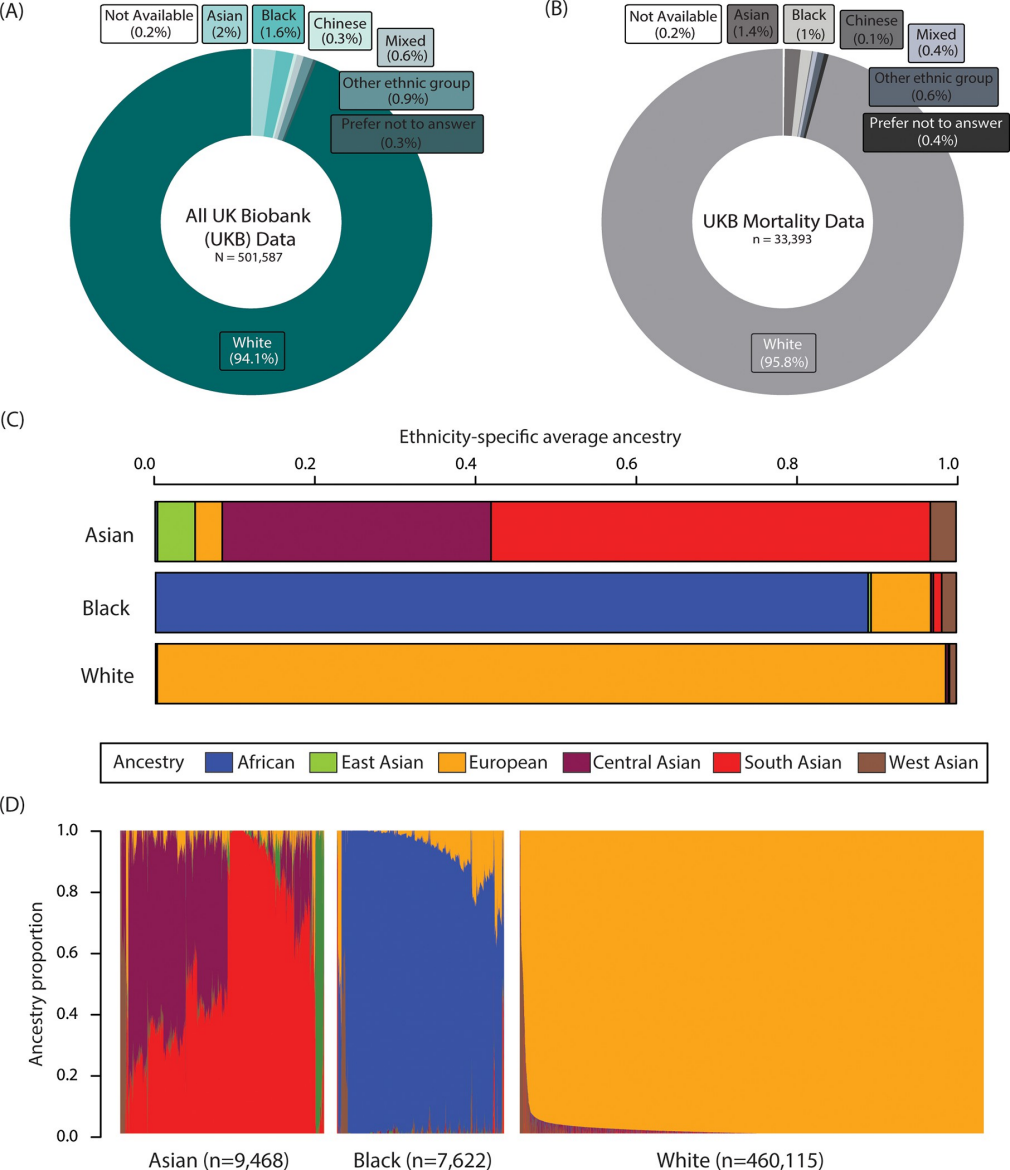
Ethnicity and genetic ancestry in the UKB. Participant ethnic group percentages for (A) the entire UKB cohort and (B) participants with mortality data. (C) Average genetic ancestry proportions for Asian, Black, and White ethnic groups. (D) Individual participant ancestry group proportions stratified by ethnicity. Continental ancestry group proportions are shown as: African (blue), East Asian (green), European (yellow), Central Asian (plum), South Asian (red), and West Asian (brown).

Three main ethnic groups were assigned based on self-identified ethnic background: White (British/Irish/Any other white background/White), Asian (Indian/ Pakistani/Bangladeshi/any other Asian background/Asian or Asian British), and Black (African/Caribbean/any other Black background). Other ethnic groups were not included in this study due to low sample size, comprising <1% of the total and dead datasets (Fig 1A and 1B). Of the 33,393 death records, Whites made up 95.82% followed by Asians with 1.37% and Blacks with 1%.

To provide ethnic group-specific models with more objective and granular ancestry information, GA inference was performed. Six GA proportions were estimated for 477,205 participants or 97.28% of the three-ethnic group dataset. Whites were predominantly of European ancestry (98.65%), followed by West Asian (0.69%) and Central Asian (0.33%) (Fig 1C). Blacks were also dominantly of a single ancestry, African (88.85%), followed by European (7.45%) and West Asian (1.82%). Asians were comparatively more admixed, however, with South Asian (54.78%), Central Asian (33.52%), East Asian (4.69%), European (3.39%), and West Asian (3.21%) (Fig 1C and 1D).

**Fig 2:**
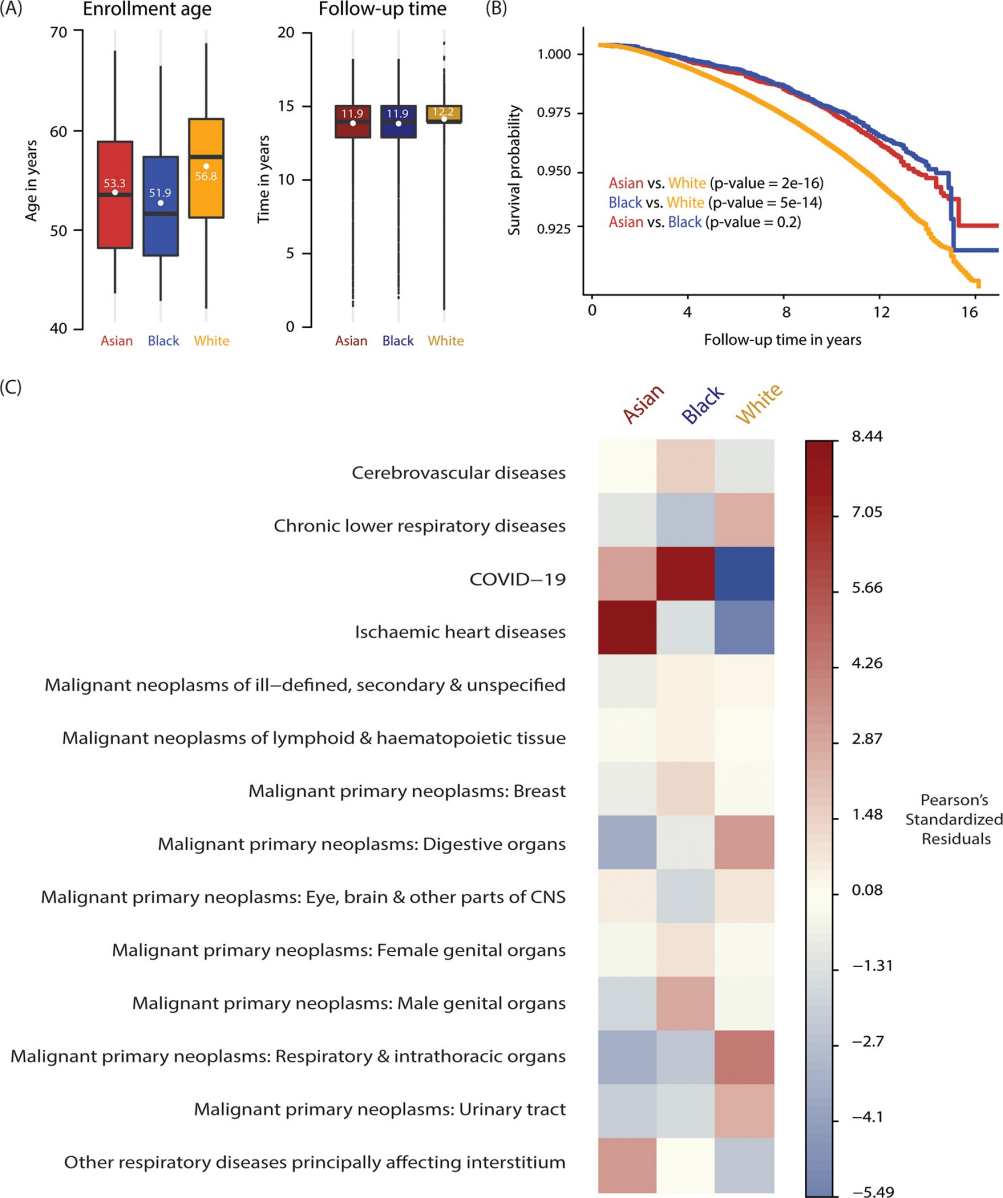
Ethnicity and mortality in the UKB. (A) Study enrollment age and follow-up time distributions for Asian (red), Black (blue), and White (yellow) ethnic groups. (B) Kaplan-Meier curves showing survival probabilities over time for Asian, Black and White ethnic groups. P-values for ethnic group pairwise log-rank test of survival curves are shown. (C) Associations between ethnicity and specific mortality causes as measured by Pearson’s standardized residuals from Chi-square test of independence.

There were observable differences in age of enrollment across the ethnic groups (i.e., Whites were enrolled at median age of 58 years, compared to Asians at 53 Blacks at 50.5), while the follow-up times were consistent with median of 12 years for all three groups (Fig 2A). Kaplan Meier (KM) survival probability curves for each ethnic group and pairwise log-rank test of difference in curves showed significant difference for Asian vs. Whites and Blacks vs. Whites (Fig 2B). Differences also existed between top causes of death and their associations across ethnic groups. Primary reasons of death from Death Registry data coded in International Classification of Diseases, Tenth Revision, Clinical Modification (ICD10) were analyzed at the block level in each ethnic group and Chi-square test of independence was performed for association between causes of mortality and ethnicity. For Asians, the top causes were ischemic heart diseases (22.27%), followed by primary malignant neoplasms or cancers of digestive organs (9.17%), while deaths from digestive cancers were most frequent for Blacks (13.43%) and Whites (15.69%) (S3 Fig). Pearson’s Chi-squared test showed significant association between top causes of mortality and ethnicity with particularly strong positive associations between ischemic heart disease and Asians (std.residual = 8.44), COVID-19 and Blacks (std.residual = 7.97), and respiratory/intrathoracic organ cancers and Whites (std.residual = 4.26) (Fig 2C). Based on the differences observed for mortality and causes of death, all downstream analyses were performed separately for each ethnic group.

### Multivariable survival analysis

#### Overall mortality

**Fig 3:**
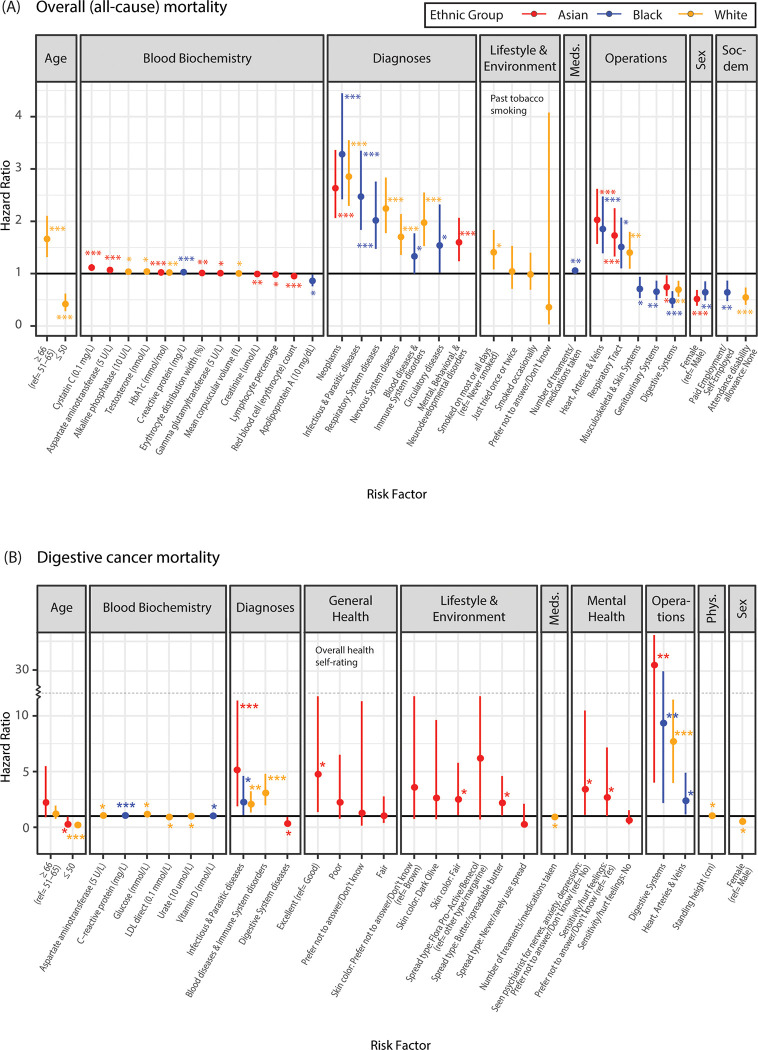
Ethnicity and mortality risk factors. Risk factor-mortality associations, as measured by Cox proportional hazard ratios (with 95% CIs), are shown for Asian (red), Black (blue), and White (yellow) ethnic groups. Significance of association measured in p-values are indicated in stars. Associations are shown for (A) overall (all-cause) mortality and (B) digestive cancer mortality. Mortality risk factor categories include: age, blood biochemistry, previous in-patient disease diagnoses (diagnoses), lifestyle and environmental measures, medications (meds.), previous in-patient operations and/or procedures (operations), sex, sociodemographic factors (soc-dem), general health, mental health, and physical measures (phys.).

Feature selected mortality risk factors (S3 Table) were analyzed in multivariable Cox-PH models and their effect sizes or hazard ratio (HR) were estimated. For overall mortality, previous in-patient diagnoses, such as neoplasms, and operations and/or procedures, such as on heart, arteries, and veins or on respiratory track, had greatest impact on overall mortality in all three groups (Fig 3A). However, some diagnoses were uniquely important or more significant to specific ethnic groups. For example, having mental and behavioral diagnoses increased relative risk of mortality by 60% in Asians (HR = 1.598, CI_95%_ = [1.236,2.066], *p* = 0.00035), while having infectious and parasitic diseases increased the risk by nearly 2.5 times in Blacks (HR = 2.472 [1.829,3.342], *p*<0.0001) (S4A Table). In contrast, operation on digestive organs including upper endoscopy and colonoscopy was the only type of in-patient procedures associated with reduced mortality risk in all three groups (HR_Asian_ = 0.743 [0.571,0.967], *p* = 0.027; HR_Black_ = 0.479 [0.345,0.665], *p*<0.0001; HR_White_ = 0.694 [0.559,0.863], *p*<0.0001) (Fig 3A). Being female was associated with reduced mortality risk by more than 36% compared to males in Asian and Black group but not in Whites. Several blood and urine biomarkers showed significant effect on overall mortality of Asians including cystatin-C (HR = 1.115 [1.051,1.183], *p* = 0.00031) and aspartate aminotransferases (HR = 1.069 [1.036,1.102], *p*<0.0001). For Blacks, increase in CRP levels (mg/L) was highly associated with increase in mortality risk (HR = 1.028 [1.013,1.043], *p* = 0.00034), while apolipoprotein (ApoA) levels (10 mg/dL) reduced mortality risk by 14% (HR = 0.862 [0.759,0.978], *p* = 0.021). Moreover, having paid employment status or being self-employed decreased mortality risk by 36% in Blacks (HR = 0.642 [0.476,0.866], *p* = 0.0038). Significant environmental and sociodemographic risk factors for overall mortality in Whites were past smoking status (smoked on most or all days; HR = 1.408 [1.081,1.833], *p* = 0.011) and receipt of disability support or allowance (none; HR = 0.545 [0.405,0.733], *p*<0.0001). In all three ethnic groups, GA informed the models but did display significant effects on overall or cause-specific mortalities.

#### Cause-specific mortality: Digestive cancers

Previous diagnoses of infectious and parasitic diseases were important risk factor in all three groups, increasing the relative risk of death by at least 2 folds (HR_Asian_ = 5.141 [2.266,11.660], *p*<0.0001; HR_Black_ = 2.253 [1.101,4.608], *p* = 0.026; HR_White_ = 2.069 [1.328,3.223], *p* = 0.0013) (Fig 3B). In addition, operation on digestive organs was the largest risk factor in all three groups, since endoscopy and colonoscopy are the main methods used in digestive cancer diagnosis (S4B Table). Unique or group-specific patterns were observed including increase in glucose levels (mmol/L) and standing height (cm) both significantly associated with increase in relative mortality risk by 18% and 3%, respectively, per unit change in Whites (HR_glucose_ = 1.175 [1.029,1.343], *p* = 0.017; HR_s.height_ = 1.034 [1.001,1.068], *p* = 0.044). Increase in CRP levels was again highly associated with increase in mortality risk for Blacks (HR = 1.053 [1.022,1.086], *p* = 0.00084). For Asians, consumption of butter as main spread type doubled the risk of digestive cancer mortality than those consuming other spread types or margarine (HR = 2.187 [1.040,4.597], *p* = 0.039). Hesitancy to answering questions (i.e., choosing “prefer not to answer” or “don’t know” instead of “yes” or “no”) related to mental state or illness, such as “have seen a psychiatrist for nerves/anxiety/depression” or “have sensitive or hurt feelings”, was associated with higher risk of digestive neoplasm mortality in Asians (HR_sensitive_ = 2.677 [1.001,7.16], *p* = 0.049; HR_psychiatrist_ = 3.408 [1.109,10.474], *p* = 0.032). Moreover, Asians who self-reporting their overall health as “excellent” showed greater risk of mortality than those indicated as having “good” health (HR = 4.759 [1.365,16.590], *p* = 0.014).

#### Cause-specific mortality: COVID-19, ischemic heart disease, and lung & bronchus cancers

For COVID-19 deaths in Blacks, males had increased relative risk of mortality by 72% compared to females (HR_female_ = 0.285 [0.108,0.750], *p* = 0.011), and history of hospitalization due to influenza and pneumonia (HR = 13.905 [1.779,108.709], *p* = 0.012) and receiving ventilation support (HR = 4.841 [1.949,12.026], *p* = 0.039) were most significant predictors of mortality for diagnosis and operations, respectively (S5A Fig; S4C Table). Similar to overall and digestive cancer mortalities, increase in CRP was associated with increased mortality risk to COVID-19 in Blacks (HR = 1.043 [1.004,1.082], *p* = 0.030). Increase in waist circumference (cm) was another highly significant risk factor unique to COVID-19 risk of death (HR = 1.059 [1.028,1.091], *p* = 0.00014). For ischemic heart disease deaths in Asians, increase in cystatin-C, being male, and past smoking (smoked most or all days) all significantly increased the mortality risk (HR_cystatinC_ = 1.194 [1.035,1.378], *p* = 0.015; HR_female_ = 0.071 [0.015,0.339], *p* = 0.00090; HR_smoking_ = 2.847 [1.461,5.550], *p* = 0.039) (S5B Fig). For deaths due to lung and bronchus cancers in Whites, having previously diagnosed chronic lower respiratory disease (HR = 1.859 [1.190,2.904], *p* = 0.0065), procedures on respiratory tract (HR = 9.069 [5.894,13.954], *p*<0.0001), and increase in alkaline phosphatase (HR = 1.073 [1.025,1.122], *p* = 0.0023) increased mortality risk, while increase in ApoA (HR = 0.896 [0.834,0.963], *p* = 0.0030) and fresh fruit and breakfast cereal intake (HR_fruit_ = 0.924 [0.877,0.973], *p* = 0.0027; HR_cereal_ = 0.956 [0.922,0.991], *p* = 0.015) lowered the mortality risk for Whites (S5C Fig).

#### Mortality risk factor interactions with ethnicity

**Fig 4:**
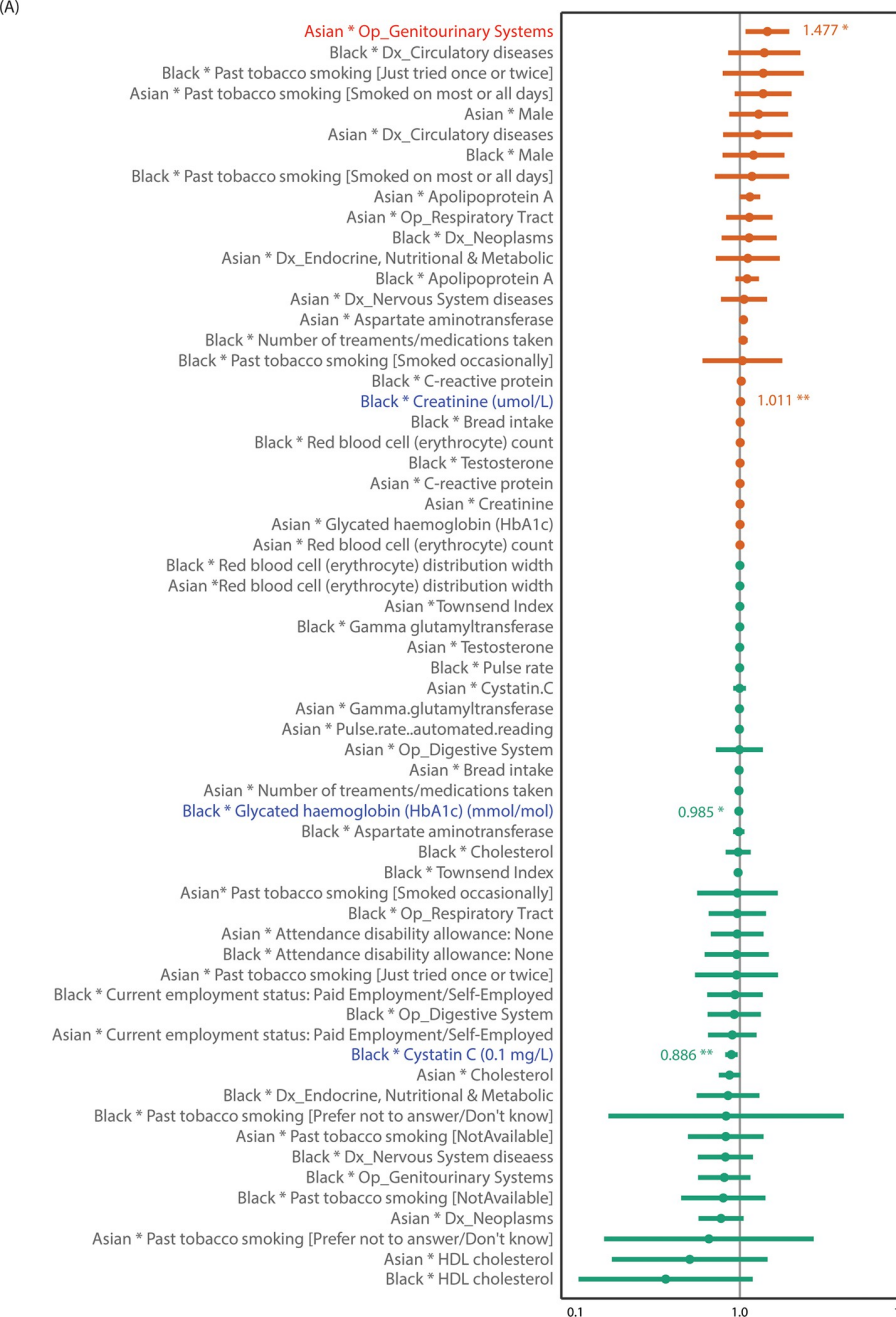
Risk factor interactions with ethnicity on overall mortality. Forest plots showing HR and 95% CI bars of ethnicity-by-risk-factor interactions tested in the two pooled sample survival models combining Whites, Blacks, and Asians for overall (all-cause) mortality. Significant interactions with Blacks (blue) and Asians (red) are highlighted with stars showing significant level (* for <0.05; ** for <0.01).

**Fig 5:**
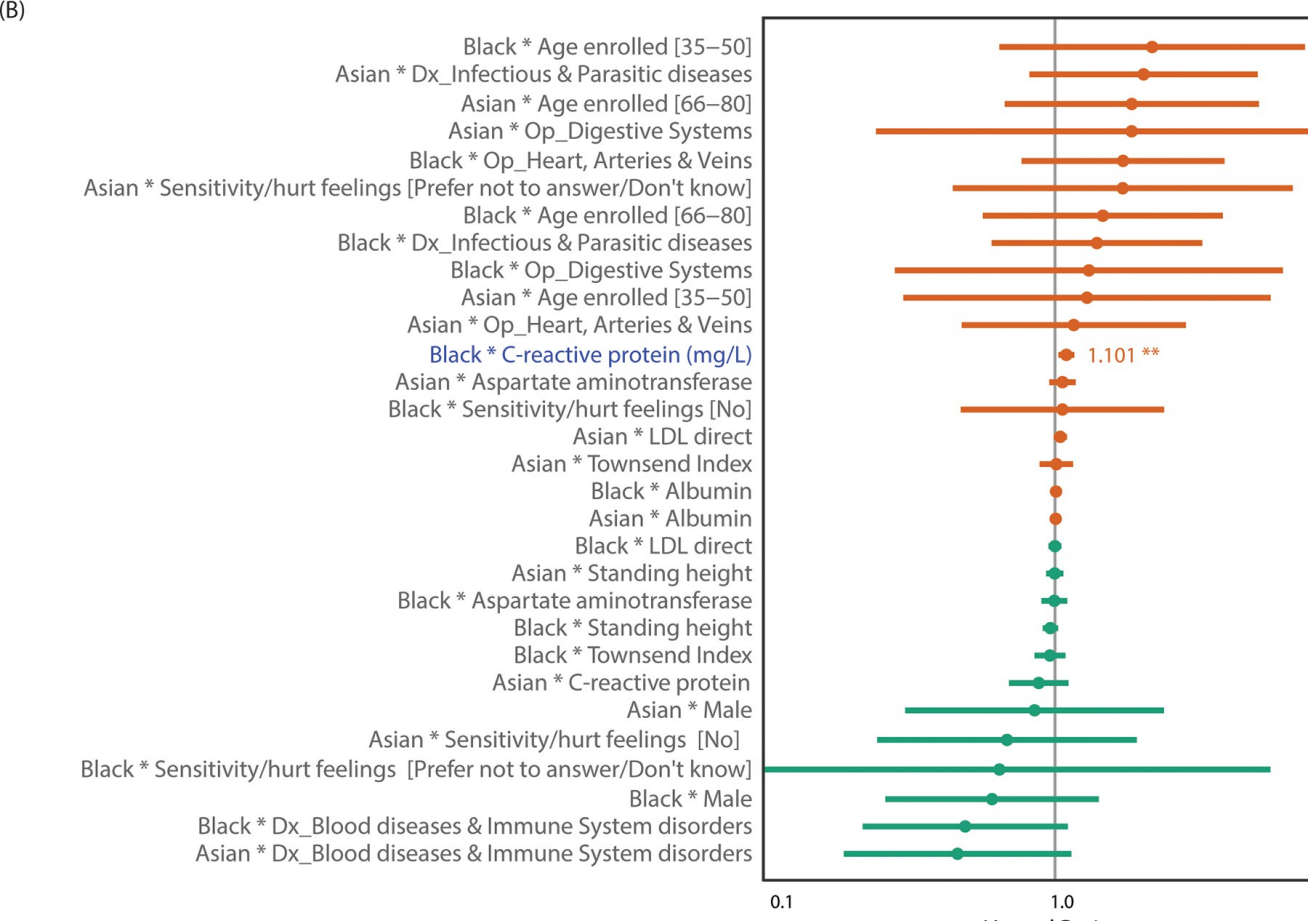
Risk factor interactions with ethnicity on digestive cancer mortality. Forest plots showing HR and 95% CI bars of ethnicity-by-risk-factor interactions tested in the two pooled sample survival models combining Whites, Blacks, and Asians for digestive cancer mortality. Significant interactions with Blacks (blue) are highlighted with stars showing significant level (* for <0.05; ** for <0.01).

Two pooled-sample multivariable survival models were tested for interactions between Asian and Black ethnic groups and 31 all-cause mortality risk factors (Fig 4) and 15 digestive cancer mortality risk factors (Fig 5). For all-cause mortality, there was one significant interaction between Asian ethnicity and operations and/or procedures on genitourinary systems (HR = 1.477 [1.084,2.014], *p* = 0.014) and three significant interactions between Black ethnicity and blood biomarkers: creatinine (HR = 1.011 [1.003,1.019], *p* = 0.0099), glycated hemoglobin (HbA1c) (HR = 0.985 [0.972,0.999], *p* = 0.0042), and Cystatin-C (HR = 0.886 [0.813,0.967], *p* = 0.0066) (S4D Table). Thus, genitourinary systems-related operations in Asians and higher creatine level in Blacks had significantly greater adverse effect on overall survival compared to their effects in the White group, while higher levels of cystatin-C and HbA1c had less adverse effect in Blacks compared to their effects in Whites. For digestive cancer mortality, one significant interaction between Black ethnicity and CRP emerged (HR = 1.101 [1.029,1.178], *p* = 0.0051), showing a greater adverse effect of increased CRP levels in Blacks compared to Whites (S4E Table).

## Discussion

In this study, we characterized mortality patterns across three largest ethnic groups in the UK and identified significant mortality risk factors for each, using group-specific feature selection and survival modeling of the UKB data. Our study demonstrated that mortality disparity exists and assessed the impact of shared and group-specific mortality risk factors for overall and other leading cause-specific mortalities per ethnicity.

Differential survival was seen between ethnic groups with Whites showing worse survival probability compared to Black and Asians in UKB. The same trend was observed in the recent analysis of death registration from England and Wales by the Office for National Statistics in the UK, which reported that Whites had higher all-cause mortality rates than other ethnic groups between 2012 to 2019 [25]. The top causes of death associated with each ethnicity also varied, delineating diseases that increase mortality and are in need of intervention for each group.

Feature selection of top risk factors and survival analysis results elucidated both general and targeted strategies for reducing mortality and disparity across ethnicities. Serious preexisting medical conditions, based on ICD10 and preventive or diagnostic OPCS Classification of Interventions and Procedures, had the greatest impact on mortalities in all three ethnic groups: neoplasms increased relative risk of overall mortality by over 2 folds, while exams of digestive organs showed a protective effect, reducing the risk of overall mortality by at least 25%. Thus, focusing on cancer prevention and surveillance methods, such as receiving endoscopic exam of gastrointestinal tract, colon, and lower bowel, may be healthful in reducing the overall mortality in the UK irrespective of ethnicity [26].

Conversely, other preexisting medical conditions and diagnoses were ethnic-specific including the mental and behavioral diseases and Asian mortality. The relative risk of mortality in Asians, who are mostly South Asians of Indian, Pakistani, and Bangladeshi origin in the UKB, increased by 60% when previously diagnosed with mental illnesses related to psychoactive substance abuse, organic mental disorders, anxiety, and depression (S5 Table). Asian participants also evaded directly answering mental health-related questions, and this observed hesitancy was significantly associated with a greater risk of mortality in Asians dying of digestive cancers. High prevalence of mental disorders and reluctance to discuss mental illness have also been reported in both India and for Asian Indian communities in the US, where the perception of mental health issues has been marred by social stigma and cultural shame, which has contributed to avoidance in psychological diagnosis and care [27–30]. Moreover, several studies have indicated that South Asian immigrants experience high rates of mental health disorders, which has been linked to reduction in general life expectancy and worse disease outcomes including cancer [31–34]. These finding underscore the importance of sociocultural factors and mental health and its significant impact on Asian mortality. Reducing stigmatization and increasing awareness of mental illnesses and access to related care represent targeted opportunities for Asians.

Several blood and urine biomarkers showed specific associations with ethnicity and mortality. CRP, a biomarker for chronic inflammation, is an important mortality risk factor for Blacks, as evidenced by the significant association between increased CRP levels and greater risk of both overall and cause-specific deaths due to COVID-19 and digestive cancers. Elevated CRP levels have been previously associated with diseases including diabetes and cardiovascular disease and were found in African Americans and Black ethnic groups at higher concentrations [35–37]. Chronic inflammation, as measured by CRP and other blood biomarkers, has been linked to physiological responses to psychosocial stressors, including exposure to discrimination [38]. Here, CRP showed a significant interaction with Black ethnicity in the pooled survival model for digestive cancer mortality, suggesting a greater adverse effect of elevated CRP in Blacks compared to Whites. This finding further reinforces the importance of CRP as mortality risk factor in the Black ethnic group. Similarly, increased levels of cystatin-C, a marker of renal function, in Asians and their overall and ischemic heart disease deaths, and glucose in Whites dying from digestive cancers, were significantly increased the risk of mortality. Glucose was found to promote invasion and metastasis of colon cancer cells, which fit with our finding as a significant risk factor for White mortality from digestive cancers [39]. HbA1c, an indicator of average blood glucose level in the last 90 days, was also associated with greater risk of overall mortality in both Whites and Asians. Meanwhile, HbA1c negatively interacted with Black ethnicity, suggesting that Blacks experience a reduced adverse effect of elevated HbA1c on their overall survival compared to Whites. Alternatively, the ‘diminished returns’ hypothesis suggests that given the presence of numerous other risk factors in the Black group, the presence or absence of individual-level exposures, such as HbA1c, are less significant for Black than White individuals [24, 40–42]. Consistent with diminished returns, absent elevated levels of specific exposures including HbA1c, Whites are expected to live longer, whereas Black mortality changes substantially less across different levels of the same risk factors. Additionally, high concentrations of cystatin-C have been linked to greater risk of heart failure and death in persons with coronary heart disease (CHD) [43]. Our results reinforce and further suggest that cystatin-C is an important overall mortality risk predictor for Asians and Asians dying of CHD. Finally, the protective effect with increased level of ApoA for Black overall mortality and White mortality due to lung and bronchus cancers also aligns with previous reporting of inverse correlation between ApoA levels and risk of developing lung, colorectal, breast, and ovarian cancers [44]. Biomarkers of this kind are routinely used to assess health and disease status and have been linked to various factors, such as genetics, dietary and behavioral, and environmental pollutants [45, 46]. Identification of measurable biomarkers with ethnic group-specific effects can inform precise strategies and potential biological targets for reducing associated mortality.

Several modifiable risk factors for mortality related to physical measures, dietary habits, and health behaviors were also identified including lessening the consumption of butter for Asians (digestive cancer), increasing fresh fruit and breakfast cereal intake and quitting smoking for Whites (lung and bronchus cancer), and lowering waist circumference for Blacks (COVID-19). Waist circumference, but not body mass index or weight, was feature selected as a significant risk factor for COVID-19 deaths in Blacks, suggesting that adiposity around the waist may be more effective predictor of COVID-19 deaths than other related body measurements [47]. These key environmental factors are modifiable and can help reduce mortality risks.

Lastly, two sociodemographic risk factors, disability living allowance for Whites and having paid or self-employment status for Blacks, impacted the overall mortality. However, other socioeconomic measure of deprivation, such as Townsend index scores, was not found to be a significant risk to ethnic-specific mortality. This is likely attributable to within-group similarities in SES of participants of same ethnicity, which may also explain the lack of significant associations with genetic ancestry proportions and ethnic-specific mortalities. Moreover, this demonstrates that the effects of individual-level risk factors highlighted in this study, intermediate and proximal, outweigh that of SES, a distal risk factor, when we independently investigated the main contributors of mortality in each ethnicity [48]. A number of proximal risk factors also showed significant interactions with ethnic groups, adding to growing evidence of the unequal effects of the same risk factors on mortality across different race and ethnicity groups [24, 40–42]. Therefore, population-specific study design that inform targeted risk factors and strategies for reducing mortalities will be critical to overcoming disparities across race and ethnicity groups.

There are several limitations to this study. First, The UKB data is sampling of people living across the UK with median age of enrollment over 50. There may be bias of results due to left truncation, since the analysis was based on participants who had survived to the late enrollment age. In addition, race and ethnicity classification varies between countries for it is a socially defined membership and self-identified based on shared heritage, culture, and social experiences. While the UK ethnic groups studied here approximately correspond to US racial groups, evidenced by similarity to genetic ancestry proportions of White and African Americans, the social experiences and potential health implications could be different [49]. Thus, the extent to which the study findings may be transferable to other countries like the US is uncertain and may require additional validation using data based on the population of interest.

## Conclusions

Our findings demonstrate the ethic differences in mortality and associated risk factors that may contribute to the observed disparities. Several measurable and modifiable blood biomarkers and environmental and behavioral factors were identified including unexpected associations between ethnic mortality disparities, mental health, and systemic stress, some of which showed differential effects on mortality across ethnic groups. These results underscore the importance of population-specific studies that can help decompose health disparities and inform targeted interventions towards greater health equity.

## Supporting information

S1 Fig Breakdown of potential mortality risk factors tested by UKB’s primary category of origin. Tree diagrams showing the breakdown of mortality risk factors considered in this study by the UKB’s primary category of origin.
(TIF)

S2 Fig Principal component analysis plots of (A) reference population groups and (B) all UKB ethnicity groups. PCA plots showing PC1 and PC2 of seven different reference populations and UKB participants used in this study by their GA and ethnic backgrounds.
(TIF)

S3 Fig Top causes of mortality for three ethnic groups (Asian, Black, White) in UKB. Relative frequency heatmap by ethnic groups and their leading primary cause of death.
(TIF)

S4 Fig Feature selection performance plot. The optimal number features were based on feature selection performance, as shown here, by selecting minimum number of top features yielding maximum average C-index across 5 repeats of 5-fold CV.
(TIF)

S5 Fig Ethnicity and cause-specific mortality risk factor forest plots. Risk factor-mortality associations, as measured by Cox proportional hazard ratios (with 95% CIs), are shown for Asian (red), Black (blue), and White (yellow) ethnic groups. Significance of association measured in p-values are indicated in stars. Individual plots for (A) Asian–ischemic heart disease, (B) Black–COVID19, and (C) White–Lung/Bronchus cancer are shown. Mortality risk factor categories include: age of enrollment, blood biochemistry, previous in-patient disease diagnoses (diagnoses), lifestyle and environmental measures, family history, previous in-patient operations and/or procedures (oper.), sex, and physical measures (phys. measure).
(TIF)

S1 Table Reference population groups from IKGP and HGDP used for genetic ancestry inference. Information on the reference populations groups and its source used for GA inference in this study.
(TIF)

S2 Table Field name and reference level mapping. Mapping and reference level information for significant mortality risk factors highlighted in this study. Category, field type, names used, and the unit information is provided.
(XLSX)

S3 Table Feature selected mortality risk factor ranking. Feature selected mortality risk factors rankings based on univariate Cox-PH modeling, random survival forest minimum depth, and average ranking of the two for (A) overall, (B) digestive neoplasm, and (C) other cause-specific mortalities.
(XLSX)

S4 Table Cox proportional hazard (PH) modeling results for three ethnic groups and overall and causes-specific mortalities. Multivariable surviving modeling results for (A) overall, (B) digestive neoplasm, (C) other cause-specific mortality, (D) interaction with ethnicity for pooled overall, and (E) interaction with ethnicity for pooled digestive neoplasm mortalities.
(XLSX)

S5 Table Top five blocks under selected ICD-10 diagnosis and OPCS4 operation chapters. Relative frequency table for top five blocks under each selected previous in-patient diagnoses and operations by mortality type and ethnic group.
(TIF)
