# Supplementary figures and images for: Ethnic disparities in mortality and group-specific risk factors in the UK Biobank
Source: PLOS Glob Public Health. 2023 Feb 23;3(2):e0001560. doi: 10.1371/journal.pgph.0001560 (PMC10021328; doi:10.1371/journal.pgph.0001560)

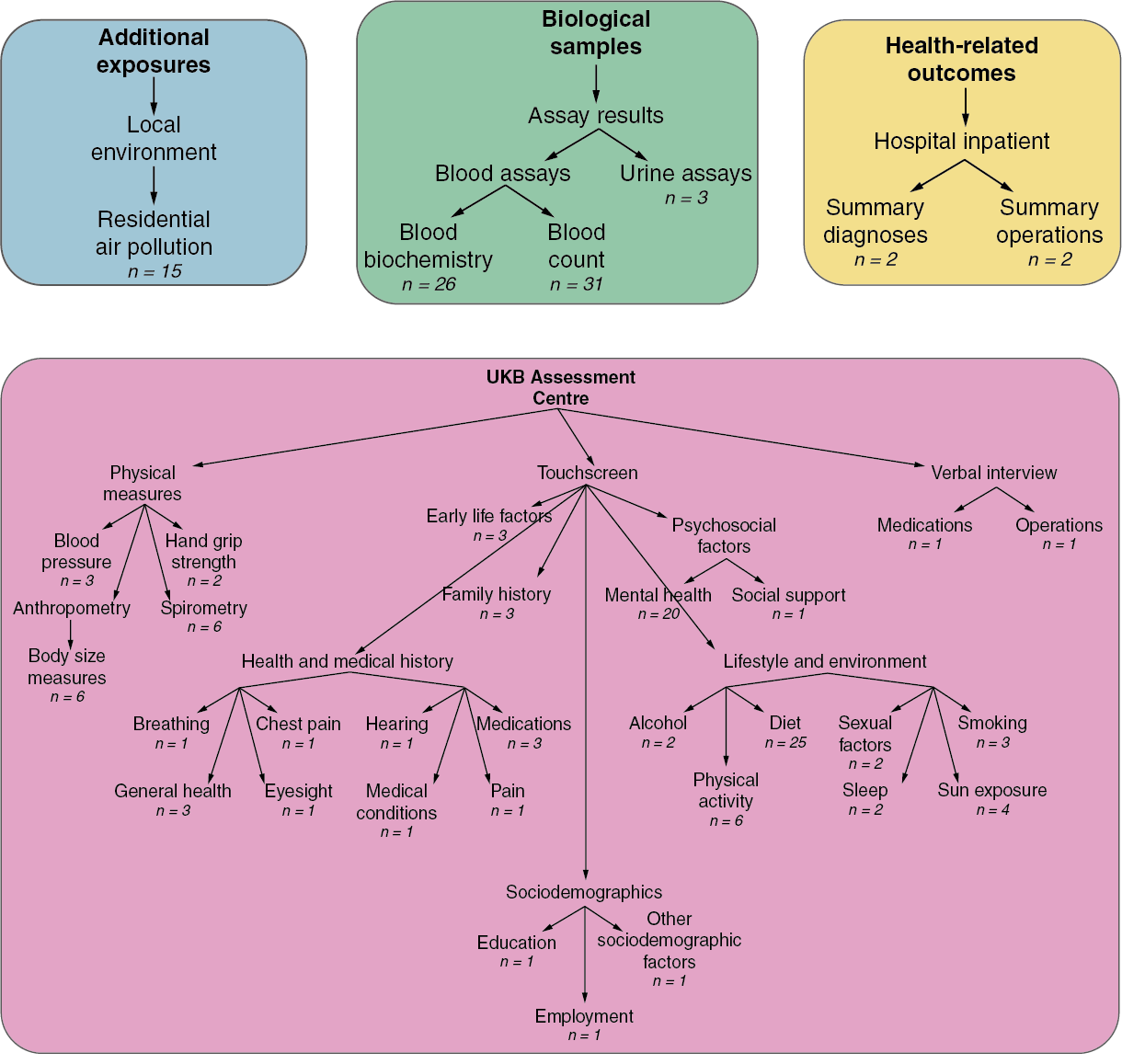

Supplement: S1 Fig — Tree diagrams showing the breakdown of mortality risk factors considered in this study by the UKB’s primary category of origin. (TIF) [file pgph.0001560.s001.tif]

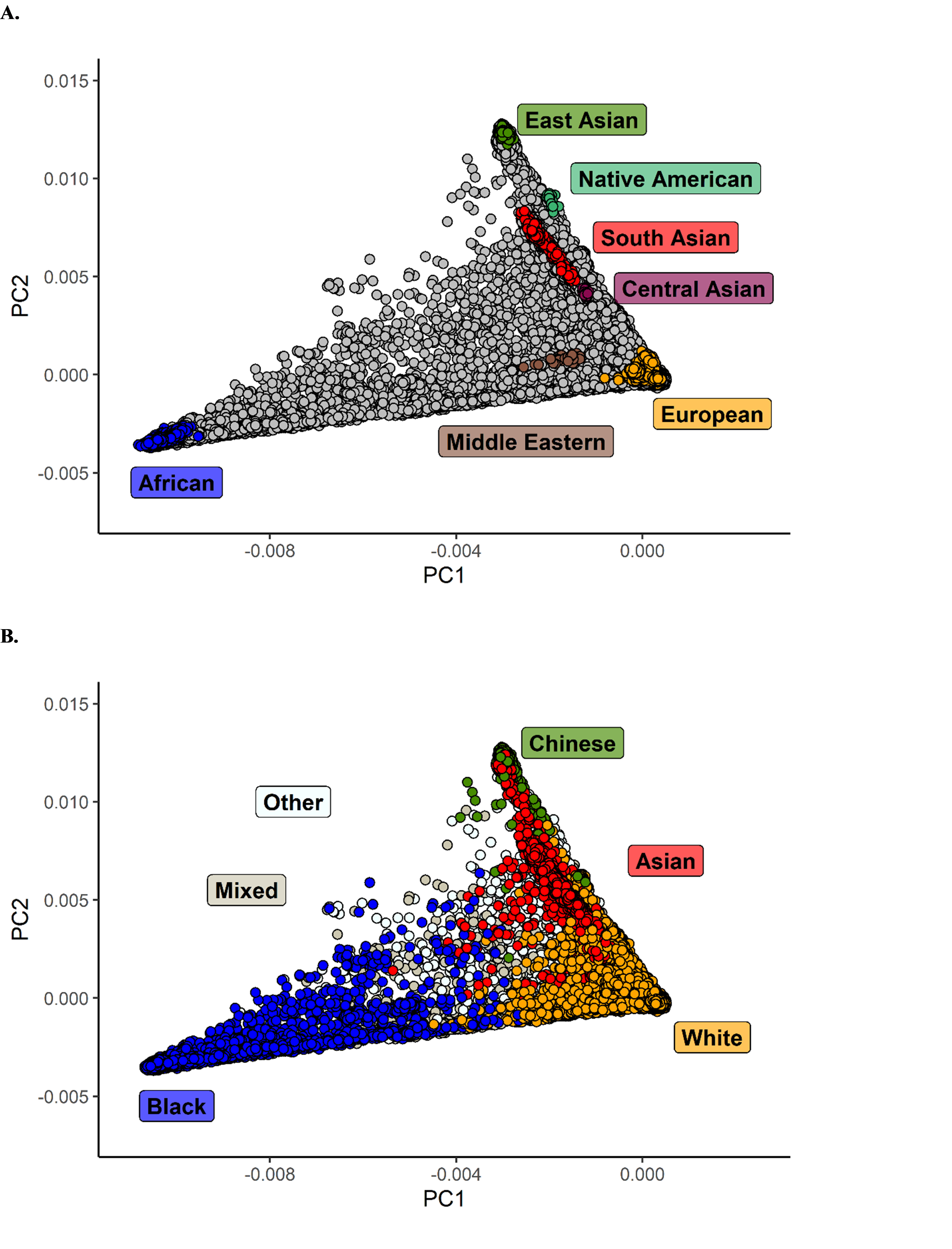

Supplement: S2 Fig — Principal component analysis plots of (A) reference population groups and (B) all UKB ethnicity groups. PCA plots showing PC1 and PC2 of seven different reference populations and UKB participants used in this study by their GA and ethnic backgrounds. (TIF) [file pgph.0001560.s002.tif]

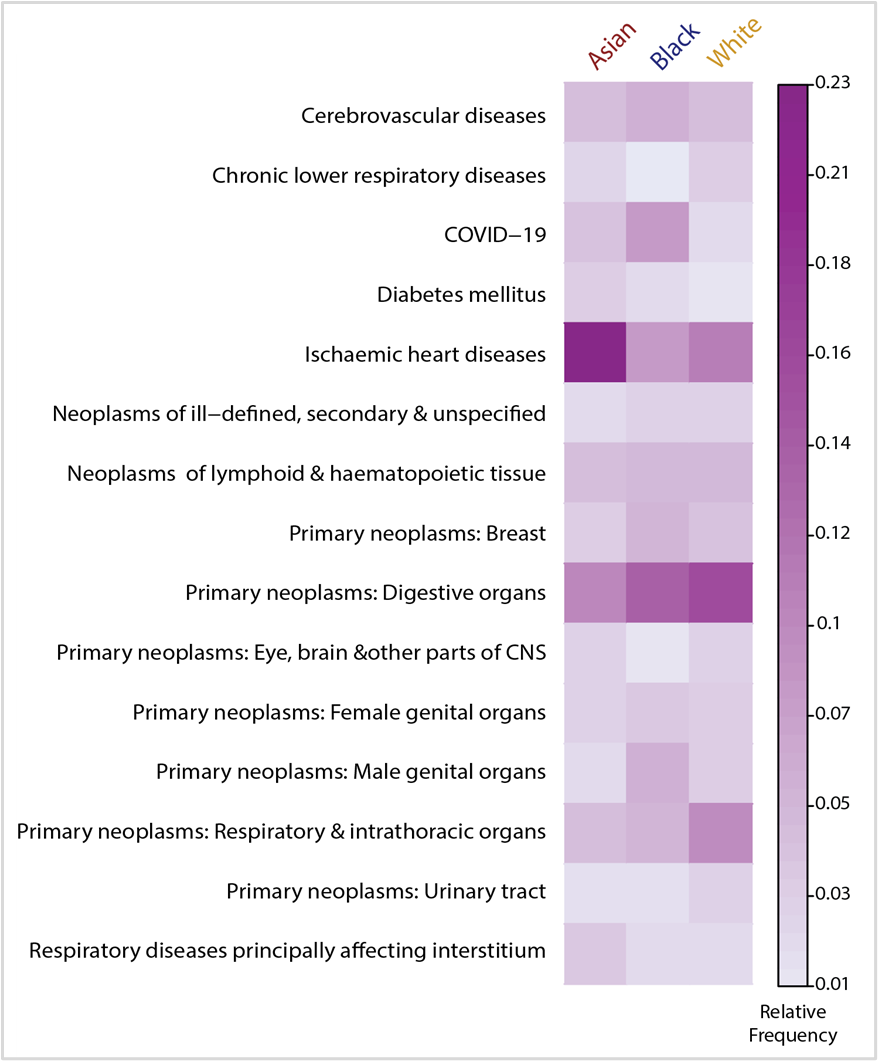

Supplement: S3 Fig — Relative frequency heatmap by ethnic groups and their leading primary cause of death. (TIF) [file pgph.0001560.s003.tif]

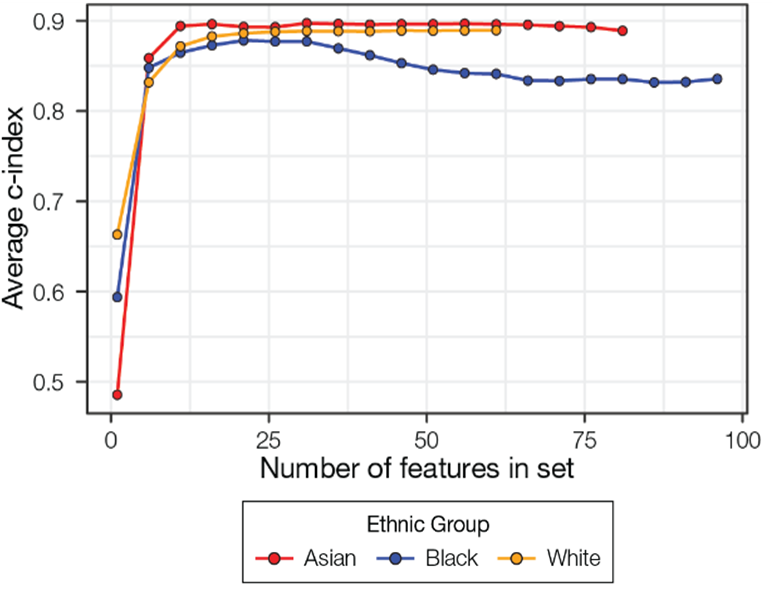

Supplement: S4 Fig — The optimal number features were based on feature selection performance, as shown here, by selecting minimum number of top features yielding maximum average C-index across 5 repeats of 5-fold CV. (TIF) [file pgph.0001560.s004.tif]

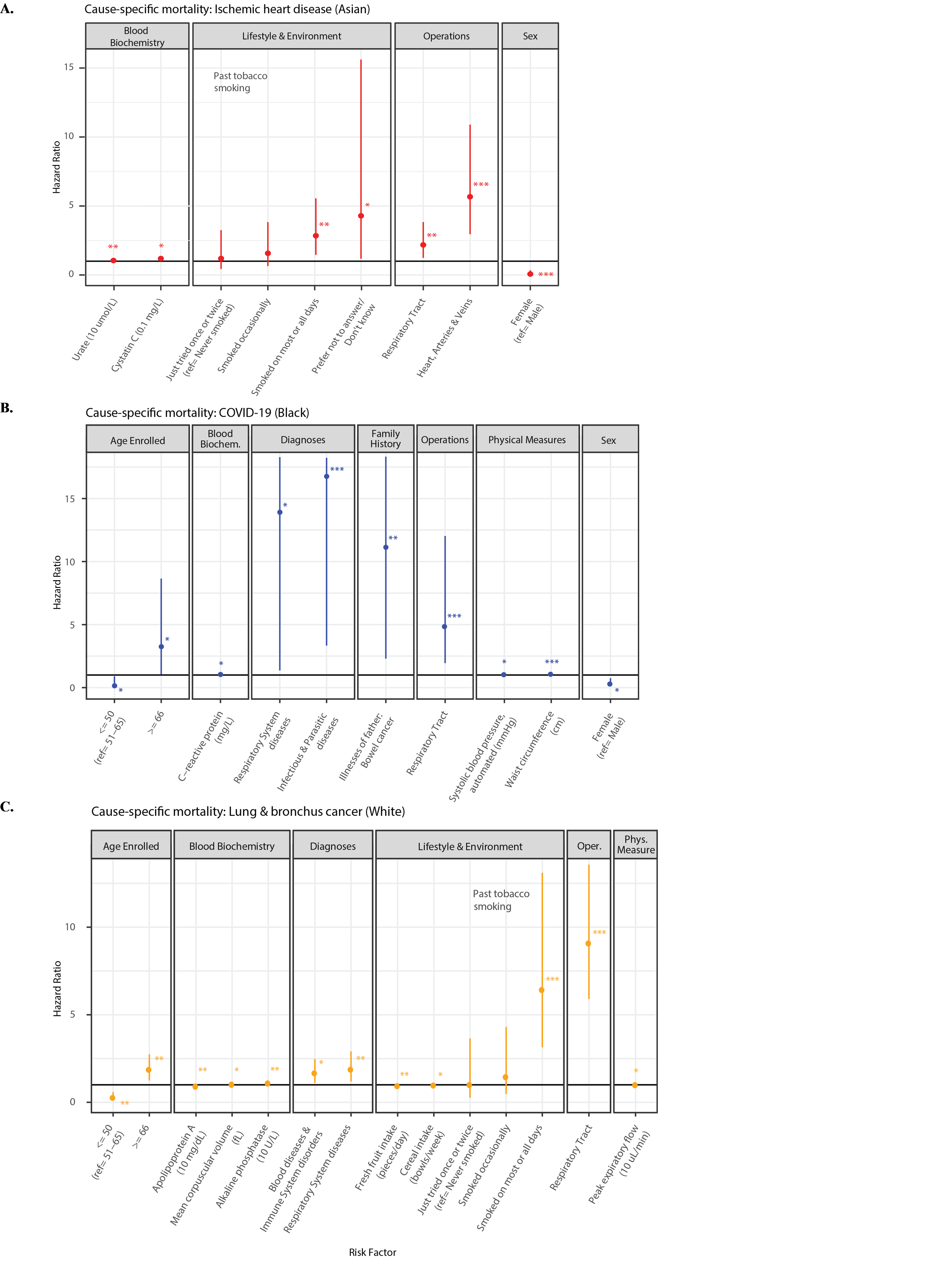

Supplement: S5 Fig — Risk factor-mortality associations, as measured by Cox proportional hazard ratios (with 95% CIs), are shown for Asian (red), Black (blue), and White (yellow) ethnic groups. Significance of association measured in p-values are indicated in stars. Individual plots for (A) Asian–ischemic heart disease, (B) Black–COVID19, and (C) White–Lung/Bronchus cancer are shown. Mortality risk factor categories include: age of enrollment, blood biochemistry, previous in-patient disease diagnoses (diagnoses), lifestyle and environmental measures, family history, previous in-patient operations and/or procedures (oper.), sex, and physical measures (phys. measure). (TIF) [file pgph.0001560.s005.tif]

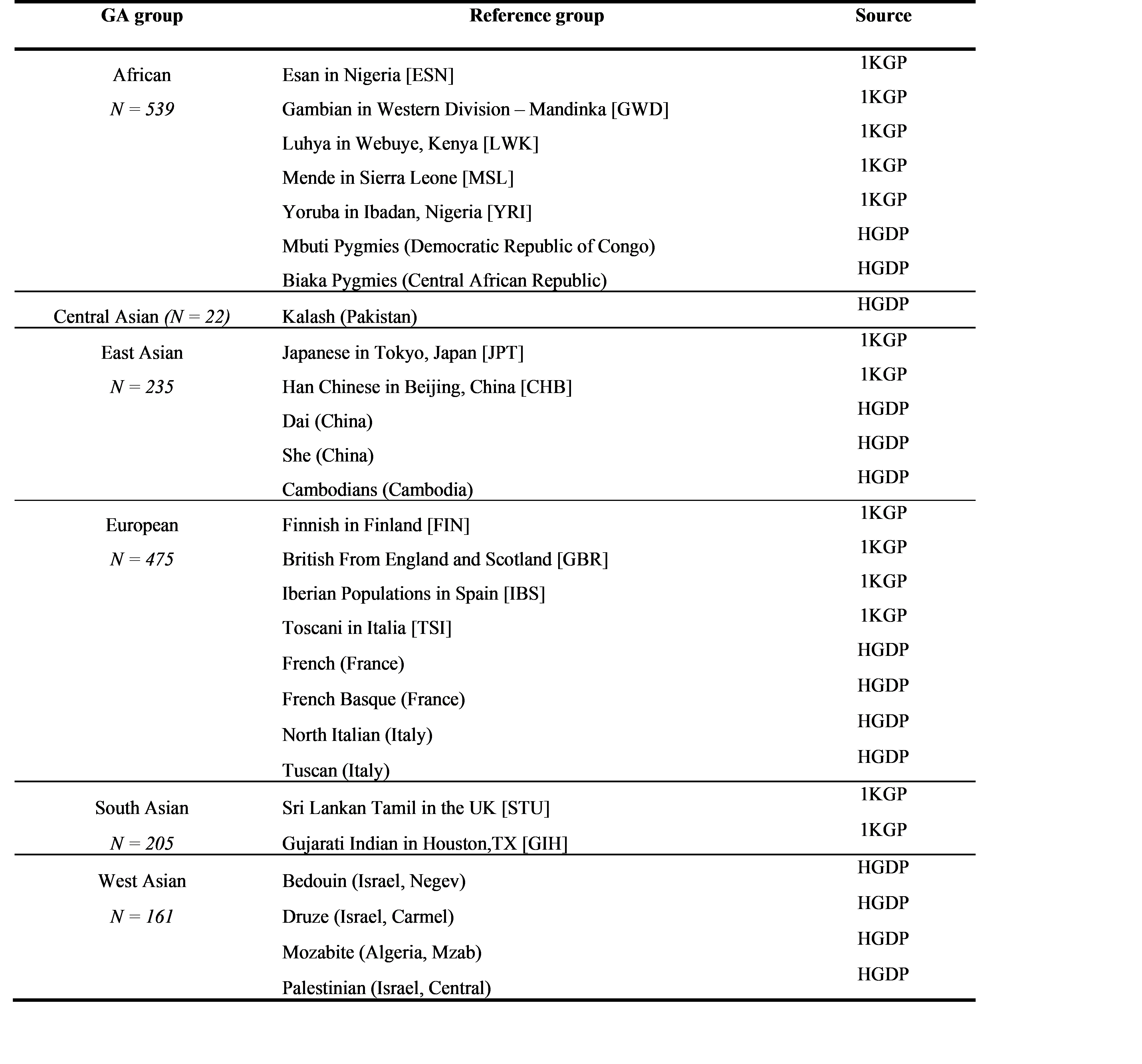

Supplement: S1 Table — Information on the reference populations groups and its source used for GA inference in this study. (TIF) [file pgph.0001560.s006.tif]

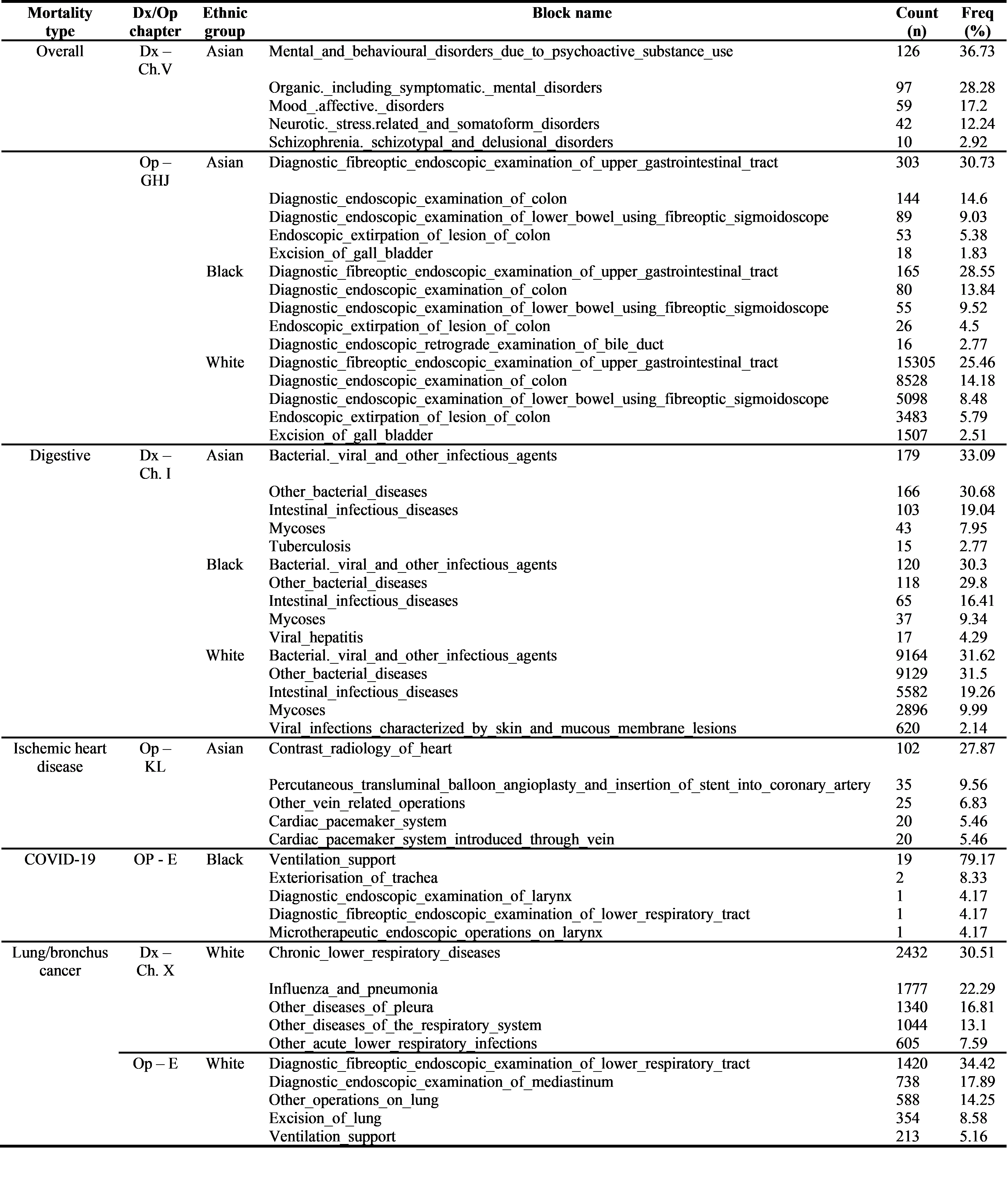

Supplement: S5 Table — Relative frequency table for top five blocks under each selected previous in-patient diagnoses and operations by mortality type and ethnic group. (TIF) [file pgph.0001560.s010.tif]
